# S48. INTER-RATER RELIABILITY OF PANSS-6 SCHIZOPHRENIA SEVERITY RATINGS OBTAINED USING THE SIMPLIFIED NEGATIVE AND POSITIVE SYMPTOMS INTERVIEW (SNAPSI)

**DOI:** 10.1093/schbul/sby018.835

**Published:** 2018-04-01

**Authors:** Pernille Kølbæk, Per Bech, Ole Mors, Christoph U Correll, Søren D Østergaard

**Affiliations:** 1 Aarhus University Hospital; 2 Mental Health Centre North Zealand, University of Copenhagen; 3 Aarhus University Hospital, Aarhus University; 4 The Zucker Hillside Hospital

## Abstract

**Background:**

Schizophrenia is a severe mental disorder requiring multimodal treatment. Monitoring the severity of schizophrenia during treatment is essential to a successful outcome. The most widely used measure of the severity of schizophrenia is the 30-item Positive And Negative Syndrome Scale (PANSS-30) obtained by the Structured Clinical Interview, SCI-PANSS, which takes approximately an hour to administer. This is too long for routine clinical use. Recently, our group extracted a 6-item scale (PANSS-6), which has shown promising psychometric properties. The scale consists of the following items: P1 - Delusions, P2 - Conceptual disorganization, P3 - Hallucinatory behavior, N1 - Blunted Affect, N4 - Passive/apathetic social withdrawal and N6 - Lack of spontaneity & flow of conversation. For now, it remains unknown whether it is possible to obtain sufficient information for PANSS-6 rating via a short and focused interview. Recently, our group developed an interview, the Simplified Negative and Positive Symptoms Interview (SNAPSI), which enables PANSS-6 rating. Field-testing at hospitals in the United States and Denmark has shown that the patient section of SNAPSI can be completed in approximately 15–25 minutes by raters who are unfamiliar with the interview and involving patients hearing the questions for the first time. The aim of the present study was to test the inter-rater reliability of PANSS-6 ratings obtained using the SNAPSI.

**Methods:**

The team of raters (five medical doctors and two psychologists) attended training sessions prior to the inter-rater reliability test. At the training sessions one rater interviewed a patient with schizophrenia using the SNAPSI, while all raters conducted PANSS-6 ratings independently. After each interview the PANSS-6 ratings were discussed until an agreement was reached. Each rater participated in at least six SNAPSI/PANSS-6 training ratings.

For the inter-rater reliability test, a total of 12 patients with a primary diagnosis of schizophrenia, currently undergoing in- or outpatient treatment at the Department for Psychosis, Aarhus University Hospital – Denmark, will be recruited. The team of raters will perform a total of at least 50 PANSS-6 ratings via SNAPSI. All raters will conduct the SNAPSI at least once. As a measure of inter-rater reliability, we will calculate the Intraclass Correlation Coefficient based on the 50 PANSS-6 ratings.

**Results:**

The results of the inter-rater reliability test will be available in January 2018 and presented at the SIRS 2018 conference.

**Discussion:**

If the results of the inter-rater reliability test are satisfactory, we will conduct a clinical validation of PANSS-6. In this study we will test whether PANSS-6 ratings obtained using the SNAPSI correspond to PANSS-6 ratings extracted from independent PANSS-30 ratings obtained using the SCI-PANSS. If this is the case, PANSS-6 ratings obtained using the SNAPSI will facilitate valid measurement-based care of schizophrenia in clinical practice.

